# Cathelicidins Modulate TLR-Activation and Inflammation

**DOI:** 10.3389/fimmu.2020.01137

**Published:** 2020-06-09

**Authors:** Maaike R. Scheenstra, Roel M. van Harten, Edwin J. A. Veldhuizen, Henk P. Haagsman, Maarten Coorens

**Affiliations:** ^1^Division of Infectious Diseases and Immunology, Department of Biomolecular Health Sciences, Utrecht University, Utrecht, Netherlands; ^2^Division of Clinical Microbiology, Department of Laboratory Medicine, Karolinska Institute, Stockholm, Sweden; ^3^Department of Clinical Microbiology, Karolinska University Laboratory, Stockholm, Sweden

**Keywords:** macrophages, dendritic cells, antimicrobial peptides, cathelicidins, LL-37, Toll-like receptors, MAMPs, DAMPs

## Abstract

Cathelicidins are short cationic peptides that are part of the innate immune system. At first, these peptides were studied mostly for their direct antimicrobial killing capacity, but nowadays they are more and more appreciated for their immunomodulatory functions. In this review, we will provide a comprehensive overview of the various effects cathelicidins have on the detection of damage- and microbe-associated molecular patterns, with a special focus on their effects on Toll-like receptor (TLR) activation. We review the available literature based on TLR ligand types, which can roughly be divided into lipidic ligands, such as LPS and lipoproteins, and nucleic-acid ligands, such as RNA and DNA. For both ligand types, we describe how direct cathelicidin-ligand interactions influence TLR activation, by for instance altering ligand stability, cellular uptake and receptor interaction. In addition, we will review the more indirect mechanisms by which cathelicidins affect downstream TLR-signaling. To place all this information in a broader context, we discuss how these cathelicidin-mediated effects can have an impact on how the host responds to infectious organisms as well as how these effects play a role in the exacerbation of inflammation in auto-immune diseases. Finally, we discuss how these immunomodulatory activities can be exploited in vaccine development and cancer therapies.

## Introduction

The discovery of penicillin in 1928 by Alexander Fleming was a major breakthrough in medicine. Ever since, the use of antibiotics saved millions of lives around the world, curing infections that were previously life threatening (1). However, due to the continuous expansion of antibiotic resistance among clinically relevant bacterial species, novel antimicrobials are urgently required to counter infections by these pathogens (2). One promising alternative to conventional antibiotics is the use of host defense peptides (HDPs), which refers to a large family of peptides with varying functions, including direct antimicrobial activity against a wide variety of bacterial, fungal and viral pathogens. Of special interest is the more recent description of the immunomodulatory functions of these peptides, which provides additional opportunities for potential clinical applications (3).

One group of HDPs that has been extensively studied in the context of their immunomodulatory activity is the cathelicidin family. This peptide family can be found in nearly all vertebrate species and has been shown to have a major impact on host responses toward various highly conserved microbe-associated molecular patterns (MAMPs). MAMPs activate the innate immune system through pattern recognition receptors (PRRs), of which Toll-like receptors (TLRs) are the most well-known receptor family. TLRs can be roughly divided into two subgroups; extracellular TLRs and intracellular TLRs, that recognize microbial membrane components and extracellular proteins or nucleic acids, respectively (4).

In this review, we aim to summarize the current knowledge on the mechanisms by which cathelicidins affect TLR activation and downstream signaling as well as how this impacts immune responses during both infections and sterile inflammation, including auto-immune responses.

## Cathelicidins

Cathelicidins belong to the family of HDPs with each cathelicidin being encoded by a single gene, consisting of four exons. Cathelicidins are translated as a pre-pro-peptide, consisting of a signal peptide on the N-terminus that directs the peptide to secretory granules, followed by the highly conserved cathelin domain and ending in the active mature peptide at the C-terminus (5). Cathelicidins, such as the human cathelicidin LL-37, are commonly secreted by neutrophils in their biologically inactive pro-peptide form and require cleavage by extracellular enzymes such as elastase or proteinase-3 to release the biologically active C-terminal peptide (6). In the human skin, proteases such as kallikrein-5 have also been shown to cleave the LL-37 pro-peptide (hCAP18) once it is secreted by epithelial cells and keratinocytes. This leads not only to release of the active LL-37 peptide, but also to many different smaller fragments, such as LL-23, LL-29, and KS-27 (7). The mature cathelicidin peptides are highly variable in both amino-acid sequence and size, which leads to considerable differences in their 3D structure. They can contain to α-helices, β-hairpins, extended structures or form cyclic peptides. Some cathelicidins are rich in specific tryptophan, proline or arginine residues, while others are arranged in short tandem repeats (8–10). Since the mature peptides are highly diverse, not all cathelicidins will have similar activities which is important to keep in mind when studying these peptides. Importantly, despite these highly diverse structures, most peptides have a characteristic amphipathic nature and a net positive charge (8, 11).

Cathelicidins are expressed in nearly all vertebrates. In some species, only one cathelicidin has been identified, like human (LL-37), mouse (CRAMP) and dog (K9CATH). Other species, like chicken, horse, pig and cattle, express multiple cathelicidins (10). The main source of LL-37 in humans are neutrophils, which store the inactive pro-peptide in their secretory granules (12) and secrete them upon activation (13, 14). However, other cell types, including lymphocytes, macrophages, epithelial cells and keratinocytes, can also produce cathelicidins (15–17). Under homeostatic conditions, cathelicidins reach *in vivo* concentrations of around 0.2–0.5 μM in the plasma (12, 18), 0.2–2.0 μM in the lung mucosa (18), 0.01–1.1 μM in sweat (19), 0–4.4 μM in ascites fluid and 4–6 μM in saliva (18). Many cathelicidins are strongly upregulated during infection due to TLR activation by MAMPs, such as LPS, LTA and flagellin (20, 21). In addition, cathelicidins can be upregulated when tissues are damaged or by exposure to specific compounds, such as vitamin D3, butyrate and PGE2 (22–25). Under extreme conditions, for example in psoriatic lesions, more than 300 μM cathelicidin can be detected (26).

While best known for their direct antimicrobial activity against a broad spectrum of bacteria (27–29), viruses (30–32), fungi (33, 34), and parasites (35, 36), it is now well-established that these peptides also have the potential to modulate immune responses in various ways. This includes regulation of neutrophil and monocyte chemotaxis (37–39), induction of chemokine expression (27, 40), skewing of macrophage polarization (41), influencing phagocytosis (27, 42–44), and regulation of both extracellular and intracellular TLR activation (27, 40, 45–49). Due to this plethora of effects, it is perhaps not surprising that the reduced expression or total lack of cathelicidins is correlated with increased risk of infection (50, 51) but also has an impact on the development of autoimmune diseases (52–55).

## Cathelicidins Inhibit The Activation of Lipid-Sensing TLRs

### Lipid-Sensing TLRs

**Figure 1:**
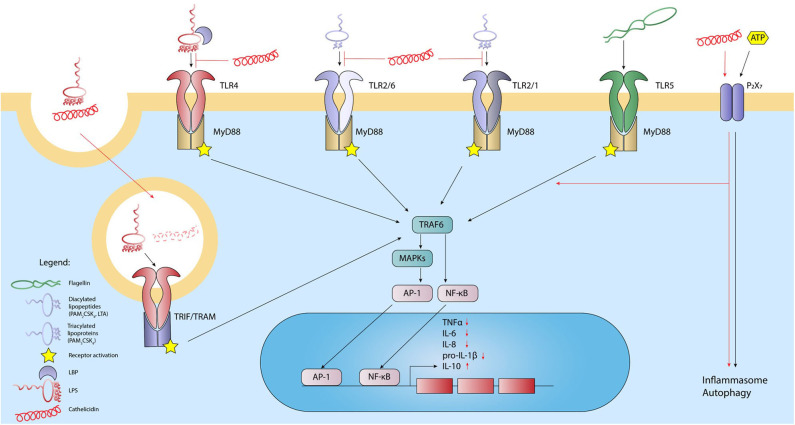
Cathelicidins inhibit the activation of lipid-sensing TLRs and modulate the response of other extracellular receptors. Activation of TLR4 by LPS, TLR1/2, or-2/6 heterodimers by LTA, and TLR5 by bacterial flagellin leads to activation of the intracellular domain. Through the adaptor MyD88 and subsequent downstream signaling through MAPKs, NFkB, and AP-1 are released and translocated to the nucleus, leading to transcription of inflammatory cytokines. LPS can also activate TLR4 located in endosomal compartments, which leads to activation through adaptors TRIF and TRAM. Additionally, P_2_X_7_ receptor responds to extracellular ATP, which leads to inflammasome formation. In general, cathelicidins inhibit lipid-induced TLR-activation and thereby the downstream inflammatory response by neutralizing lipid MAMPs. LPS-cathelicidin complexes can be taken up, after which the cathelicidins are degraded to allow LPS-induced TLR4 stimulation. Finally, cathelicidins can signal through the P_2_X_7_ receptor by which the peptides can stimulate inflammasome formation and autophagy. *Black lines: normal signaling; red lines: effects exerted by cathelicidins*.

Extracellular TLRs are important in the detection of bacteria-derived lipid-containing molecules. Detection of such lipids is often the first step in the initiation of an immune response against many bacterial pathogens. Bacterial lipid-containing molecules that can activate TLRs include lipopolysaccharides (LPS) from the Gram-negative bacterial outer membrane (TLR4), lipoteichoic acids (LTA) from the Gram-positive bacterial cell wall and diverse di- and tri-acylated bacterial lipoproteins (TLR1/2/6). During activation, TLRs form homo- or heterodimers that are the basis of the TLR receptor complex. However, various co-receptors, such as MD-2 and CD14 have been shown to improve ligand detection by TLRs. Upon stimulation, TLR4 forms a receptor complex consisting of a TLR4 homodimer and two MD-2 proteins (4, 56, 57). The expression of the CD14 co-receptor can further enhance LPS detection and cellular responses. The soluble LPS-binding protein (LBP) can further act as a chaperone by extracting LPS from the bacterial membrane or bacterial-derived outer membrane vesicles and delivering it to the TLR4 receptor complex. TLR2 on the other hand forms heterodimers with either TLR1 or TLR6 (58, 59). These TLR2 heterodimers are responsible for the recognition of a variety of MAMPs, including LTA, di- and tri-acylated bacterial lipoproteins such as the highly common Braun lipoprotein in *E. coli*, lipoarabinomannan from mycobacteria, zymosan from fungi and hemagglutinin from measles viruses. In addition, synthetic lipoproteins based on these natural ligands, such as the di-acylated Pam2CSK4 (TLR2/6) and tri-acylated Pam3CSK4 (TLR1/2), are commonly used as TLR2 ligands for *in vitro* studies. Similar to TLR4 activation, expression of CD14 further increases the detection efficiency of TLR1/2/6 receptor complexes (56). Both TLR4 and TLR1/2/6 signal via the MyD88-dependent pathway, which ultimately leads to activation of NF-κB and AP-1 and thereby to the secretion of pro-inflammatory cytokines (56, 60). Importantly, TLR4 can also be present in endosomal compartments where activation can lead to TRIF-mediated signaling pathways, leading to the production of anti-inflammatory cytokines like IL-10 and type I interferons, predominantly IFN-β (61) (Figure 1).

### Cathelicidins Inhibit LPS-Induced TLR4 Activation

Due to the fact that cathelicidins were initially described for their antimicrobial and membrane disruptive activity, many studies have focused on elucidating how cathelicidins interact with bacterial membranes or specific membrane components, such as LPS and LTA. Through these studies, it has become clear that cathelicidins are attracted to the bacterial membrane via electrostatic interactions between the negatively charged lipids in the bacterial outer membrane and the positively charged peptide (62). Indeed, loss of negatively charged phosphate-groups in the LPS core-region, for example due to mutations in LPS-controlling genes such as *PhoP/PhoQ* or *PmrB/PmrA*, reduces the susceptibility of Gram-negative bacteria to host defence peptides, like cathelicidins (63). While LPS-cathelicidin interaction may be important for eliciting antimicrobial activity, it was first shown in 1994 by Hirata et al. (64), that the 18 kDa rabbit cationic protein (CAP18) also exerts LPS-neutralizing activity, which drastically inhibits the inflammatory responses toward LPS both *in vitro* and *in vivo* (65–67). Later studies showed that this LPS-cathelicidin interaction resulted in reduced TLR4 activation and was not limited to hexa-acylated *E. coli* LPS (68–72), but was also observed in the context of penta-acylated *P. aeruginosa* LPS and the tetra-acylated *P. gingivalis* LPS (68, 70, 73). For several cathelicidins, including the human LL-37, it has now been shown that direct complex formation between LPS and cathelicidins plays an important role in preventing the binding of LPS to the TLR4 receptor complex, thereby reducing immune activation (66, 74–79). More detailed studies on LL-37 showed that binding of *E. coli* LPS occurs in a two-step process, with strong ionic interactions being followed by lower affinity interactions that are more dependent on entropic forces, such as interaction between hydrophobic regions of LPS and LL-37 (79, 80). That LPS neutralization is at least partially dependent on this ionic interaction is underlined by the fact that citrullinated LL-37 loses its ability to reduce the LPS-induced activation of macrophages (81, 82). Importantly, cathelicidins can influence LPS-induced TLR4 activation at different stages. Chicken cathelicidin-2 (CATH-2) and LL-37 have been shown to directly penetrate the bacterial membrane and bind to membrane lipids during the bacterial killing process (79, 80). Furthermore, several cathelicidins are able to bind LPS which was already bound to LBP or are capable of reducing the LPS concentration on the host cell surface (67, 76, 83, 84), suggesting competition with cell surface receptors (Figure 1). Important to note is that the mature cathelicidin peptides are highly diverse, which explains why some cathelicidins exert a strong inhibitory effect, while others do not seem to affect TLR4 activation by LPS at all (27). Similarly, cleavage of cathelicidins, which can alter the overall peptide charge or structure, can influence the LPS binding as well. For example, LL-23, a cleaved biological variant of LL-37 containing 23 amino acids, is unable to neutralize LPS (85), whereas the 31 amino acid long LL-31 is still able to inhibit the LPS-induced TLR4 activation (68).

While the interaction between cathelicidins and LPS is important for the regulation of TLR4 activation, several studies have suggested more indirect TLR4 regulation by cathelicidins. LL-37 pre-incubation, for instance, still leads to a reduction of the LPS-induced immune response *in vitro*, albeit to a lesser extent compared to LPS-LL-37 co-incubation (71). Furthermore, in human monocytes, LPS-mediated p50/p105 as well as TNF induced protein 2 expression were strongly inhibited by LL-37, in contrast to the much milder effects of LL-37 on for instance RELB, CCL4 and CXCL1 (71, 72). Similarly, LPS stimulation of bone marrow-derived macrophages from CRAMP knockout mice results in enhanced IL-10 production compared to stimulation of wildtype macrophages. However, no difference in TNF or MIP-2 production was observed between wildtype and CRAMP knockout cells (86). This selective influence on TLR4 activation could be the result of regulating the expression or functions of signaling molecules downstream of TLR4. The murine cathelicidin CRAMP for instance, reduced MyD88 synthesis and impaired the interaction between MyD88 and IRAK in murine macrophages upon LPS stimulation. In addition, CRAMP inhibited the phosphorylation of p38 and ERK downstream in this cascade, leading to a strong reduction of TNF production (86). Similarly, LL-37 was shown to inhibit the LPS-induced translocation of the NF-κB subunits p50 and p65, also resulting in a strong reduction of TNF (71, 87) and reduces the LPS-induced upregulation of TREM-1 by MyD88 (88). However, as these studies co-incubated cells with LPS and cathelicidins, it is difficult to assess to what extent these effects are just the result of reduced TLR4 activation due to the blocking of receptor activation. Nevertheless, it has been suggested that LL-37 can interact with intracellular GAPDH, an important enzyme in the glycolysis pathway, which subsequently promotes MAPK activation and chemokine expression (71). Such effects on MAPK activation can also have an impact on LPS-induced signaling pathways, which also use MAPK as intermediate signaling molecules. However, more research is needed to clarify to what extent these indirect regulatory effects of cathelicidins influence activation of TLR4 as well as other TLRs (Figure 1).

While most studies have clearly shown an inhibitory effect of cathelicidins on LPS-induced immune activation, there are also indications that in specific cases the interaction between LPS and cathelicidins can lead to enhanced cellular activation. This effect was first shown by Shaykhiev et al. (89), where LL-37-LPS complexes were shown to be taken up more efficiently *in vitro* by human bronchial epithelial cells, which subsequently led to enhanced intracellular TLR4 activation and increased IL-6 production (89). Similarly, a human adenocarcinoma colonic epithelial cell line also responded with an enhanced inflammatory response toward LPS-LL-37 complexes compared to LPS by itself (24) (Figure 1). Nevertheless, increasing the cellular uptake of LPS does not always lead to an enhanced response. For instance, cathelicidin-mediated uptake of LPS was also observed in cultured human liver sinusoidal endothelial cells; however, this did not lead to an altered immune activation in these cells (90).

### Cathelicidins Inhibit LTA-Induced Activation of TLR1/2/6

Whereas the effects of cathelicidins on LPS-induced TLR4 activation are very well-studied, the influence of cathelicidins on lipid-induced TLR1/2/6 activation is less well-known. Nevertheless, several cathelicidins were shown to inhibit LTA- or Pam3CSK4-induced TLR1/2 activation and Pam2CSK4-induced TLR2/6 activation. This includes LL-37-mediated inhibition of TNF and IL-6 release in LTA-stimulated PBMCs and dendritic cells, as well as inhibition of LTA-induced TNF release in macrophages by several cathelicidins from different species (27, 72, 91). However, similar to the inhibition of LPS-induced activation, not all cathelicidins are able to reduce TLR1/2/6-activation or might be less effective (27, 68, 72, 75). Like LPS-neutralization, the mechanism by which cathelicidins inhibit TLR2 has also been associated with direct interaction between cathelicidins and the TLR2 ligands. Using isothermal titration calorimetry as well as competition assays, both chicken CATH-2 and human LL-37 were shown to bind LTA and Pam3CSK4 directly (77, 79, 92), albeit with a lower affinity compared to their binding to LPS (77, 78). In addition, citrullinated LL-37 loses its ability to inhibit LTA-induced activation of macrophages (81), indicating that interaction with LTA, similar as with LPS, is dependent on ionic interactions. Despite the observed interaction between LL-37 and LTA, LL-37 is not able to inhibit TLR2 activation on all cell types. In human primary bronchial cells, co-incubation of LL-37 enhanced the Pam3CSK4-induced expression of IL-8 and IL-6, while no effect of LL-37 was observed in relation to LTA-induced keratinocyte activation (93). This indicates that the function of cathelicidins might differ in the context of different cell types. In addition, as TLR2 and TLR4 signaling pathways share many downstream signaling molecules, it is likely that the indirect effects of cathelicidins on downstream TLR signaling pathways related to TLR4 activation will also play a role in the cathelicidin-mediated regulation of TLR2 activation, although no proof has been provided for this yet (Figure 1).

## Cathelicidins Enhance The Activation of Nucleic Acid-Sensing TLRs

### Nucleic Acid-Sensing TLRs

Foreign nucleic acids from invading viruses, as well as several bacteria, are sensed by several intracellular PRRs. These include several DNA- and RNA-receptors in the cytoplasm, as well as specific TLRs (TLR3, -7, -8, and-9) expressed in endolysosomal compartments (94). Depending on the localization of a pathogen as well as the stage of infection, receptors at these different cellular compartments can be activated. For instance, viruses are obligate intracellular parasites, meaning they rely completely on host cells for their replication and survival and replicate their genome within the host cytoplasm. Alternatively, viral nucleic acids can be found extracellularly as well in apoptotic particles, which can be engulfed by host cells and thereby end up in TLR-containing endolysosomes (95). In contrast to extracellular TLRs, which can rapidly respond to ligands in the extracellular environment, several barriers exist for the activation of intracellular TLRs by nucleic acids. Firstly, due to the many nucleases present in the extracellular environment, most free extracellular nucleic-acids are degraded before cells have the opportunity to respond (96, 97). Secondly, because the nucleic acid sensing TLRs are located intracellularly, cells have to actively endocytose the DNA or RNA for it to reach its complementary TLR receptor (98, 99).

TLR9 recognizes unmethylated CpG-containing DNA motifs in bacterial genomic DNA and viral double stranded DNA (dsDNA). The unmethylated CpG-containing DNA motifs in bacterial DNA are mimicked by synthetic CpG oligodeoxynucleotides (ODN), which are widely used in experimental systems (94, 100, 101). TLR21 is the avian equivalent of TLR9, which also recognizes CpG-ODN (102). TLR7 and -8 are highly homologous to each other due to gene duplication and recognize viral single stranded RNA (ssRNA), RNA from bacteria such as group B Streptococci and possibly siRNAs as well. The synthetic agonists for TLR7 and -8 are antiviral nucleoside analogs such as R848 and imiquimod. TLR3 recognizes viral double-stranded RNA (dsRNA), which is mimicked by the synthetic analog polyinosinic-polycytidylic acid [poly(I:C)]. TLR3 might also respond to some ssRNA viruses, most likely during the replication phase when they copy their RNA (56, 94, 100, 101, 103).

Like most extracellular TLRs, TLR7, -8, and -9 signal through the MyD88-dependent signaling pathway, resulting in the secretion of proinflammatory cytokines. In addition, activation of the highly expressed TLR7 and -9 in plasmacytoid DCs (pDCs) results in high levels of type-I interferons, like IFN-α, which is important during anti-viral responses. TLR3 on the other hand activates the TRIF-TRAF pathway in a MyD88 independent manner, which leads to IFN-β secretion (94, 101, 103).

### Cathelicidins Promote Nucleic Acid Stability and Endocytosis

**Figure 2:**
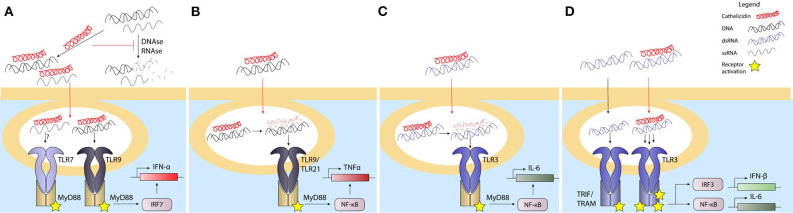
Cathelicidins influence the response to nucleic acids depending on the cell type. Extracellular nucleotides are an inflammatory signal through various pathways. Phagocytosis or endocytosis of DNA and/or RNA is required for their delivery to endosomal compartments leads to activation of TLR3 by dsRNA, TLR7/8 by ssRNA, and TLR9 by bacterial or self-DNA. Activation of the TLR response leads to activation of the NFkB and/or IRF transcription factors, an subsequently to inflammatory cytokine production. Extracellular DNAses and RNAses degrade the nucleotides, which prevents uptake by phagocytic cells and reduces TLR activation in the endosomal compartements. Cathelicidins can bind to extracellular DNA/RNA to stabilize it and prevent it from degradation by DNAses or RNAses. The uptake of DNA/RNA-cathelicidin complexes is enhanced and this complex formation enhances TLR9 and possibly TLR7 activation in pDCs **(A)**. The uptake of DNA-cathelicidin complexes by macrophages **(B)** or RNA-cathelicidin-complexes by bronchial epithelial cells **(C)** is enhanced as well, but the cathelicidin is degraded in order to DNA or RNA to activate their respective TLRs. In keratinocytes, the dsRNA-cathelicidin complex is transported to the endosome and enhances the TLR3 stimulation **(D)**. *Black lines: normal signaling or route; red lines: effects exerted by cathelicidins*.

Cathelicidins have been found to play an important role in improving the detection of nucleic acids by cells. First of all, the positively charged cathelicidins can directly interact with the negatively charged DNA or RNA through ionic interactions, which protects it from degradation by DNAses and RNAses that are abundantly present in the extracellular environment (48, 49, 98, 104, 105). Through this interaction, cathelicidins can stabilize DNA and RNA released from damaged or dying cells as well as DNA released by bacteria, for instance during the process of biofilm formation. Once nucleic acids are bound by cathelicidins and protected from degradation, cathelicidins can assist in improving the uptake of DNA by different cell types, such as macrophages, dendritic cells and B cells (48, 49, 106, 107). While this increase in uptake could theoretically be the result of increased DNA stability, increased uptake has also been observed in the context of short DNA oligos with a phosphorothioate backbone, which are resistant to DNAse degradation due to a sulfur group replacing an oxygen group on the DNA-backbone (48, 107). Furthermore, it has been shown that the positively charged peptide can act as a bridge between the nucleic acids and proteoglycans on the cell surface, which appears to be involved in the lipid-raft-mediated uptake of these cathelicidin-nucleic acid complexes (105) (Figure 2A).

The ability of cathelicidins to increase DNA stability and enhance DNA internalization is also important during NETosis, the process by which neutrophils undergo cell death by expelling their DNA into a neutrophil extracellular trap (NET) to trap invading microbes. LL-37-DNA complexes for instance, are formed within NETs produced by neutrophils that are infected by mycobacteria. These LL-37-covered NETs, still containing mycobacteria, are more efficiently internalized by macrophages, which allows the macrophages to kill the mycobacteria in lysosomal compartments in an LL-37-dependent manner (108). Cathelicidins are also commonly found within NETs under other circumstances (104, 108, 109), where they can both contribute to the antimicrobial activity of the NET, as well as prevent the NETs from being degraded by bacterial nucleases (104).

### Cathelicidins Influence TLR Responses To Nucleic Acids Depending on the Cell Type

#### Plasmacytoid Dendritic Cells (pDCs)

pDCs play an important role in many inflammatory processes, which include wound healing, antiviral and antibacterial responses, but also autoinflammatory responses (49, 110–113). Within all these processes, their main role is the production of high quantities of IFN-α, which is produced upon activation of the endosomally located TLR7 by ssRNA or TLR9 by DNA (113). pDCs were the first cell type in which LL-37 was shown to enhance DNA-induced IFN-α responses in a TLR9-dependent manner (49). Shortly after, a similar finding was done in the context of ssRNA-LL-37-complexes, which enhanced IFN-α production in pDCs in a TLR7-dependent manner (98). While both these processes depend on the stabilization of nucleic acids by LL-37 to allow endocytosis, it was recently shown that the structural organization of DNA-LL-37-complexes is another important step in TLR9 activation. Schmidt et al. showed that DNA, when bound to LL-37, forms columnar complexes where the spacing between the DNA strands is related to the structure of the LL-37 molecules. This spacing created by LL-37 is crucial, as it approximates the width of the TLR9 ectodomain (114, 115). This ensures that the TLR9 molecules bound to the DNA do not interfere with each other, as the LL-37-mediated spacing leaves enough room for binding of other TLR9 molecules to parallel DNA strands. Furthermore, it was shown that the outside of the TLR9 ectodomain, which is not in contact with the DNA bound in the binding pocket, can interact with an adjacent DNA strand, which improves the binding affinity of the whole DNA-complex (114). Overall, the compactness of the DNA-induced by the binding with LL-37 provides an optimal spatial arrangement for the DNA to bind a high number of TLR9 receptors, which boosts the downstream signaling and IFN-α release (Figure 2B). However, similar to the indirect effects of cathelicidins on TLR4 activation, LL-37 could also play a more indirect role in TLR activation in pDCs. In these cells, autophagy is needed to deliver viral TLR7 ligands to compartments containing TLRs in order to induce an antiviral response (116). While LL-37 has not yet been shown to directly activate autophagy in pDCs, it has been shown to boost autophagy in other phagocytic cell types. Vitamin D3 for instance, a potent LL-37 inducer, triggered autophagy in human macrophages in an LL-37-dependent manner by downregulation of the PI3K/Akt/mTOR pathway (117–119), which improved the intracellular killing of *Mycobacterium tuberculosis* (119).

#### Macrophages, Conventional Dendritic Cells and B Cells

Besides pDCs, other cell types, such as macrophages (120, 121), conventional DCs (cDCs) (122, 123) and B cells (124–126), express nucleic acid recognizing TLRs. However, in contrast to pDCs, these cells do not produce IFN-α upon nucleic acid sensing, but mostly signal through MyD88-dependent proinflammatory signaling pathways, mainly involving MAPK and NF-κB (126, 127). B cells show an increased TLR9 activation upon stimulation with DNA-LL-37-complexes, which results in enhanced surface expression of activation markers CD40 and CD86, as well as increased production of IL-6 and IgG (107, 128). In human cDCs, which express TLR8 but not TLR9, ssRNA-LL-37 complexes increase the surface expression of CD80 and CD86 activation markers as well as production of IL-6 and TNF. Interestingly, IFN-α, derived from pDCs activated by ssRNA-LL-37-complexes, can further enhance the activation of cDCs by these same complexes (98).

Similar to B cells, macrophages express TLR9 in endosomal compartments and induce a proinflammatory response that includes TNF production upon DNA detection. However, where LL-37 was shown to improve responses in B cells, it has a very limited ability to enhance activation of macrophages toward DNA when tested in murine RAW264.7 macrophages. Alternatively, cathelicidins from several other species, including equine CATH-2, chicken CATH-2 and porcine PR-39, but not murine CRAMP, were able to enhance TNF responses in these cells (27). Importantly, while TLR9 activation in pDCs was shown to be dependent on the sustained complex formation between DNA and LL-37 (114), chicken macrophage activation by chicken CATH-2-DNA complexes depends on the actual release of cathelicidin from the DNA within the endosomal compartment (48). This release was the result of peptide degradation due to endosomal proteases and was a requirement for TLR21 activation. Interestingly, while exogenous CRAMP appears unable to enhance DNA-induced macrophage responses, endogenous CRAMP expression improves DNA-induced macrophage activation by upregulating TNF, IL-6 and IL-12p40 production, likely due to direct interaction between DNA, CRAMP and the endosomal TLR9 (129) (Figure 2C). While macrophage responses toward RNA-LL-37 complexes are less well-studied, some studies show that stimulation of RAW264.7 cells or alveolar macrophages with RNA-LL-37 complexes results in reduced IL-6 expression, which indicates that this complex formation actually has an inhibitory effect on the activation of TLR3 in these cells (130–132).

#### Keratinocytes and Epithelial Cells

Keratinocytes are crucial in the protection against skin infections and are a major source of LL-37 during both infections as well as wound-healing processes (133–136). The secretion of LL-37 by these cells strongly contributes to direct antimicrobial activity in the skin and enhances bacterial internalization by the keratinocytes (133). Besides acting as an antimicrobial factor, LL-37 can also influence nucleic acid detection by keratinocytes in several ways. First of all, whereas keratinocytes normally only express low levels of TLR9, LL-37 strongly induces the expression of this TLR in a dose-dependent manner, thereby increasing the capacity of keratinocytes to respond to endocytosed DNA (137–139). Secondly, expression of LL-37 during infection or skin damage gives LL-37 the opportunity to interact with host-DNA or -RNA released from the damaged tissue and influence the cellular uptake of these nucleic acids. Interestingly, while keratinocytes have an increased DNA uptake when DNA is complexed with LL-37, this DNA does not end up in endosomes, but in the cytoplasm. This alternative localization could be the reason why TLR9 cannot be activated by LL-37-DNA-complexes in keratinocytes, while consecutive stimulation with LL-37 and DNA does lead to a strong type I IFN response, possibly due to the increased expression of TLR9 induced by LL-37 (139). Nevertheless, while LL-37 promotes cytoplasmic uptake of DNA and many cytoplasmic nucleic acid receptors exist, such as the inflammasome-activating DNA receptor AIM2, the cytoplasmic localization of LL-37-DNA-complexes does not lead to activation of AIM2 nor does it activate the inflammasome-mediated release of IL-1β. The lack of AIM2 activation could potentially be caused by steric hindrance by LL-37, which might prevent the binding of self-DNA to AIM2 (140, 141) and thereby could play a role in the prevention of autoimmunity. While the studies mentioned here have mostly focused on the interaction between LL-37 and host-DNA, the upregulation of TLR9 expression could of course also influence the detection of bacterial DNA released passively by dying bacteria or actively during the programmed cell death during bacterial biofilm formation (142, 143). In contrast to LL-37-DNA complexes, complexes of LL-37 with dsRNA are in fact capable of reaching endosomal compartments in both human epidermal keratinocytes and human bronchial epithelial cells, which results in the activation of TLR3 (130, 131, 144, 145). TLR3 activation by RNA-LL-37-complexes depends on different processes in both cell types. In keratinocytes, complex formation between LL-37 and the dsRNA provides an RNA structure where the intercalating LL-37 provides optimal spacing between RNA molecules to bind a higher number of TLR3 molecules per RNA molecule, enhancing IFN-β and IL-6 production, with a similar mechanism as LL-37-DNA-mediated activation of TLR9 in pDCs. Human bronchial epithelial cells on the other hand require the dissociation of LL-37 from the LL-37-RNA-complex to activate TLR3, which is caused by a decrease in pH and protease activation in endolysosomal compartments. Degradation of LL-37 then allows the RNA to bind to the TLR3 receptor, which is reminiscent of the mechanism by which TLR21 is activated by CATH-2-DNA complexes in chicken macrophages (130, 131) (Figure 2D). Together, all these studies demonstrate both the complexity by which cathelicidins can influence nucleic acid-sensing as well as the different requirements that TLR activation by RNA/DNA-cathelicidin-complexes has depending on the cell type and species investigated.

## Limited Effects of Cathelicidins on TLR5 Activation

While the effects of cathelicidins on the previously described TLRs are well-studied, the influence of cathelicidins on TLR5 activation remains less well-understood. TLR5 detects the conserved flagellin protein present in the flagella of Gram-negative bacteria and its activation leads to pro-inflammatory cytokine production via MyD88- and NF-κB-signaling pathways (56). Some studies have shown that LL-37 enhances the flagellin-induced IL-8 secretion by adult human keratinocytes (93, 146), which was depended on P_2_X_7_ receptor signaling and Scr/Akt pathway activation (146). In human bronchial epithelial cells, co-incubation of LL-37 and flagellin resulted in an increased IL-8 and IL-6 secretion (93), regulated via the PI3K/p38 pathway (147). On the other hand, LL-37 had no or a slightly inhibitory effect on the flagellin activation in human dendritic cells (91), macrophages (81), PBMCs (68, 87), or TLR5-tranfected HEK cells (77) (Figure 1). This shows that the influence of cathelicidins on TLR5 is highly dependent on the cell type, although more research is required to understand the mechanisms underlying these differences.

## Cathelicidins Activate The Inflammasome Via P_2_X_7_

Another innate immune mechanism involved in sensing microbe- or damage-related signals involves the inflammasome. The formation of the inflammasome complex can be triggered by a diverse set of environmental stimuli, including ATP, cytokines and TLR ligands, and might be affected by cathelicidins as well. Inflammasome activation and signaling results in the conversion of pro-IL-1β and pro-IL-18 into mature IL-1β and IL-18. ATP is one of most common ligands studied in this respect, triggering the inflammasome formation via the P_2_X_7_ receptor, leading to the activation of caspase-1 and thereby the cleavage of pro-IL-1β to active IL-1β (148). However, this P_2_X_7_-mediated inflammasome activation can also be induced by other ligands, such as LL-37. This has for instance been shown by LL-37 treatment of LPS-activated monocytes or stimulation of macrophages with both LL-37 and *P. aeruginosa*, which in both cases leads to P_2_X_7_-dependent IL-1β release (149, 150). In addition, NET-associated LL-37 has been found to activate caspase-1 in a P_2_X_7_ receptor dependent fashion in macrophages, leading to IL-1β and IL-18 release (151), which provides yet another function for LL-37 in NETs.

## The Influence of Cathelicidins on TLR Activation in Health and Disease

### Cathelicidin-Mediated TLR Regulation Balances the Inflammatory Response to Bacterial Infection

#### Cathelicidin-Mediated TLR Regulation *in vitro*

Cathelicidins are capable of reducing the inflammatory response of the immune system by inhibiting LPS- or LTA-induced TLR activation. This reduction is dependent on the direct interaction between cathelicidins and these lipid-containing molecules. Until recently, these effects were mostly studied in the context of purified TLR ligands and little was known about how cathelicidins affect TLR signaling in the context of a complete bacterium. However, some recent studies are now shedding some light on how cathelicidins balance inflammation in the context of whole bacterial cells. For instance, human adenocarcinoma colonic epithelial cells produce higher amounts of LL-37 upon activation, which prevents internalization of the enteric pathogenic Gram-negative bacterium *S. typhimurium*. Alternatively, knockdown of LL-37 increases *Salmonella* invasion in enterocytes and allows for more efficient immune evasion by these bacteria due to lower TLR4 expression and a reduced IL-1β response (152). While reducing the invasion and internalization of *Salmonella* in colonic epithelial cells, LL-37 enhances the clearance of *Mycobacterium avium subsp. paratuberculosis* (MAP), a bacterium causing chronic diarrheic intestinal infections in domestic and wild ruminants, by increasing bacterial uptake in murine macrophages. In addition, macrophage treatment with LL-37 suppresses TLR2 upregulation and thereby the production of tissue-damaging inflammatory cytokines released during MAP infection (153), as well as during *A. fumigatus* infection (154).

**Figure 3:**
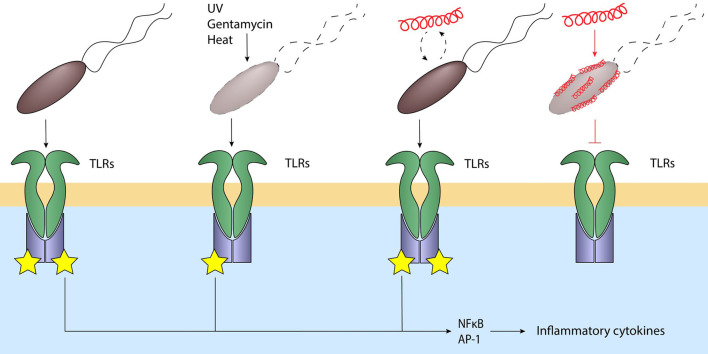
Cathelicidins balance the immune response to bacteria. Live bacteria activate various TLRs through their MAMPs, leading to NFkB and AP-1 activation and inflammatory signaling. Bacteria killed by for example UV, heat or gentamycin still activate these TLRs. Addition of cathelicidins to viable bacteria does not inhibit TLR activation; however, cathelicidin-killed bacteria or addition of cathelicidins to non-viable bacteria strongly inhibits TLR activation. This silent killing reduces the inflammatory response and thereby the subsequent inflammatory tissue damage when the bacteria are already killed and are no longer a threat. *Black lines: normal signaling; red lines: effects exerted by cathelicidins*.

Besides the ability of cathelicidins to regulate TLR expression they can also influence bacterial-induced TLR activation through direct interaction with these bacteria. Importantly, the ability of cathelicidins to regulate bacterial-induced TLR activation directly is highly dependent on bacterial viability. For instance, when cathelicidins such as human LL-37, chicken CATH-2 or porcine PMAP-36 are co-incubated with heat-inactivated *E. coli* or *P. aeruginosa*, they strongly reduce macrophage responses against these bacteria by blocking TLR2 and TLR4 activation through direct interaction with the lipoproteins and LPS normally activating these TLRs. However, when these cathelicidins are co-incubated with live *E. coli* or *P. aeruginosa*, no inhibition is observed as long as these peptides remain below bactericidal concentrations. Importantly, when instead bactericidal concentrations are used, inhibition of macrophage activation can be observed again. Alternatively, using cathelicidins that lack antimicrobial activity, but possess LPS-neutralizing activity, such as the canine K9CATH, it was shown that these peptides can in fact reduce macrophage activation in the context of killed bacteria, but not in the context of viable bacteria (73, 77, 92). This viability-dependent regulation of TLR activation provides an elegant way for the host to respond to bacterial molecules only when needed. At the start of an infection, activation of the immune system leads to the production and release of cathelicidins and cytokines from both macrophages and neutrophils at the site of infection. These cathelicidins will target and fight the bacteria to reduce the bacterial burden at the site of infection. During this phase, cathelicidins will only be able to neutralize the LPS- and lipoprotein-induced inflammatory responses against already killed bacteria, while still allowing a response against any remaining viable bacteria. This leads to a balancing act, where a reduction or increase of viable bacteria, i.e., a reduced or increased threat, also leads to a corresponding reduced or enhanced inflammatory response. Therefore, this cathelicidin-mediated regulation based on bacterial viability could be an important factor in maintaining a proportional inflammatory response based on the present bacterial threat and thereby limiting excessive inflammation which can lead to unwanted tissue damage (Figure 3).

#### Cathelicidin-Mediated TLR Modulation *in vivo*

While the strong antimicrobial potential of cathelicidins has driven the *in vivo* testing of these peptides, many studies have also investigated how these peptides influence immune activation during infection. A common tool for testing the effects of cathelicidins on infection and inflammation *in vivo* is the *clnp*-null mouse model, which lacks the expression of the only murine cathelicidin CRAMP. Using this model, it has been shown that mice lacking cathelicidin expression were more prone to necrotic skin infection caused by Group A *Streptococcus* (155), *P. aeruginosa* infection of the cornea (50), *S. aureus*-induced endophthalmitis (156), cecal-ligation and puncture-induced sepsis (157), dextran sulfate sodium (DSS)-induced colitis (158), *K. pneumoniae*-induced lung infection (159), caerulein-induced experimental acute pancreatitis (160) and meningitis-induced by intracerebral injection *S. pneumoniae* (161, 162). In general, these CRAMP-deficient mice suffered from a higher bacterial load, increased proinflammatory cytokine production and increased tissue damage. In addition, two studies also reported an increased influx of neutrophils in *clnp*-null mice (50, 162). These findings indicate the importance of cathelicidins not only in reducing the pathogenic burden during infections, but also their importance in limiting inflammation. Similarly, alternative models relying on exogenous treatment with cathelicidins, or transgenic overexpression of LL-37, show comparable results. For instance, intravenous administration of LL-37 in a cecal-ligation and puncture-induced sepsis mouse model improved the survival of these mice and reduced the bacterial load in the blood and peritoneum. In addition, LL-37 reduced the levels of several proinflammatory cytokines in the peritoneal fluids as well as in the serum, despite causing an increase in NET formation by neutrophils (163, 164). In a similar fashion, intratracheal treatment of *A. fumigatus*-instilled mice with exogenous LL-37, as well as transgenic overexpression of LL-37, causes enhanced fungal clearance, reduced lung damage and less proinflammatory cytokine production (154). Taken together, all these studies show a potent antimicrobial and anti-inflammatory role for cathelicidins during both bacterial and fungal infections. However, despite the cathelicidin-related anti-inflammatory effect seen in many of these studies, it remains difficult to distinguish if these anti-inflammatory effects can be partially caused by direct TLR inhibition or by alternative immunomodulation or that the effects can be explained by a lower bacterial burden due to the antimicrobial activity of these cathelicidins.

In an effort to separate these effects, several studies have now used different types of inactivated bacteria to investigate the direct anti-inflammatory role of cathelicidins *in vivo*. For instance, inflammatory responses induced by intratracheal administration of heat-inactivated *P. aeruginosa* or *S. aureus* isolates in mice can be inhibited by co-administration or post-administration of chicken CATH-2 (73, 165). In addition, intratracheal administration of chicken CATH-2-killed *P. aeruginosa* induces less neutrophil influx and inflammatory cytokine production compared to either administration of heat-killed or gentamicin-killed *P. aeruginosa* (73). These findings indicate that it is likely that the reduction in inflammatory markers observed upon treatment of a bacterial infection with cathelicidins, is not only the result of a reduced bacterial burden due to antimicrobial activity but is indeed also affected by direct inhibition of immune activation by cathelicidins.

### Nucleic Acid-Cathelicidin Complexes Can Lead to Autoimmune Disease

Cathelicidins have been extensively described for various protective functions that are beneficial for host survival. These functions include their direct antimicrobial activity against Gram-positive and Gram-negative bacteria (27, 74, 166), inhibition of viral replication (132) or direct antiviral activity (167), promotion of wound healing (110) as well as their ability to modulate immune responses (15, 37, 71), which has been shown to protect against excessive inflammation (77). However, their ability to improve nucleic acid detection may also lead to the onset of various autoimmune diseases.

The pathways leading to excessive inflammation in autoimmune diseases are often complex and involve numerous cell types. For SLE, psoriasis and diabetes, these processes most likely start with some type of tissue damage that initiates TLR signaling and autoimmune inflammation (49, 54, 168). In diabetes, cell death of the insulin producing β-cells of the pancreas initiates a cascade that leads to more cell death and subsequently more inflammation (54). In SLE and psoriasis, anti-DNA and -RNA antibodies can be found that most likely are the result of DNA released upon tissue damage and play an important role in the exacerbation of these diseases (168, 169). In 2007, Lande et al. were first to link these processes to cathelicidins by describing how LL-37 enhances DNA-induced inflammation in psoriatic skin lesions (49). In these lesions, the release of DNA and RNA from damaged tissue binds LL-37 (49, 98), which is expressed at extremely high concentrations (up to 300 μM) by either keratinocytes or neutrophils under these conditions (26). These complexes then stimulate pDC-derived IFN-α production that subsequently drives the activation of cDCs and T cells, which in turn exacerbate the tissue damage (98). In addition, enhanced activation of B cells by DNA-LL-37 complexes also increases the production of anti-DNA antibodies (128). In diabetes, the complex formation of these anti-DNA antibodies with DNA and CRAMP triggers pDC activation, which again leads to high IFN-α production. These high levels of IFN-α in turn increase T cell activation and thereby autoreactivity against pancreatic β-cells (54). Other complexes that can increase auto-inflammation include RNA-LL-37 complexes. These trigger TLR8-mediated cytokine production and can induce neutrophil NETosis in psoriatic skin (170). Similarly, anti-RNA immunocomplexes were shown to activate neutrophil NETosis in SLE, which results in the release of additional NET-derived DNA into the extracellular environment (168). Importantly, as these NETs are coated with both LL-37 and anti-DNA antibodies, they can serve as new ligands for pDC activation and thus IFN-α production, which leads to a further exacerbation of the inflammatory response (169).

### Cathelicidins as Adjuvant for Vaccination

Vaccination strategies aim to induce a modest immune response against one or more specific pathogens, in order for a host to be able to respond with a humoral response toward such pathogens when they are encountered later in life. To induce such a specific immune response, vaccination therapies require one or more antigens in the form of whole live, inactivated or attenuated viruses or bacteria, or alternatively, specific microbial components, such as outer membrane vesicles or specific viral or bacterial proteins. However, as not all antigens can stimulate the immune system sufficiently to build an immune memory, adjuvants, including several TLR agonists, are commonly used to improve the strength of the immune response during vaccination. Since cathelicidins can modulate these TLR responses, their possible role as adjuvant during vaccinations has been investigated in various studies. For instance, intranasal vaccination with attenuated pseudorabies virus (PRV), complexed with CpG-DNA and a bactenecin-derived innate defense regulator (IDR) peptide, resulted in an enhanced protection of piglets (171). Similarly, the combination of an IDR peptide and CpG-DNA as adjuvants for a pertussis toxoid vaccine improved *in vitro* DC maturation, cytokine production and expression of surface activation markers, while also enhancing *in vivo* antigen presentation and specific IgG1 and IgG2a antibody titers (172, 173). While the mechanisms behind these improved responses are hard to discern, improvement of DNA-induced TLR9 responses by these peptides could very well play a role in this. On the other hand, cathelicidins have also been shown to improve vaccination responses toward various viral pathogens in the absence of CpG-DNA as an adjuvant. Intramuscular or intranasal administration of piglets with inactivated porcine reproductive and respiratory syndrome virus (PRRSV) microparticles complexed with an IDR peptide or LL-37 enhanced the response toward the antigens *in vitro*; however, *in vivo* only little improvement in vaccination efficacy was observed (132). In addition, intranasal vaccination of mice using a nanoparticle-based vaccine for an H1N1-ovalbumin influenza virus, also benefits from IDR peptides as adjuvant, which together with c-di-AMP, induced a strong humoral and cellular immune response (174). Furthermore, subcutaneous vaccination of mice with the HPV E7 epitope of human papillomavirus (HPV) using CRAMP as adjuvant, reduced HPV-induced tumor growth (175). Finally, besides their effect on anti-viral vaccinations, IDR peptides were also shown to improve vaccination efficiency against other types of pathogens. This includes intravenous administration of an IDR peptide as an adjunctive therapy for an oral administered anti-malarial therapy, which strongly enhanced the protection against late-stage malarial infection in mice (176). Alternatively, using an IDR peptide as adjuvant resulted in a balanced increase in IgG1 and IgG2a antibody titers upon subcutaneous vaccination of beef calves, using a mix of *Mycoplasma bovis* subunits and IDR peptide. However, in this last study it is unclear whether this is due to the addition of the IDR peptide, since a control without IDR peptide is missing (177). Together, these results indicate the potential usefulness of cathelicidins and other similar host defense peptides in vaccination therapies; however, more detailed studies will be required to discriminate the contribution of direct immunomodulatory activities from the TLR-modulatory activities of these peptides in such vaccination therapies.

### Cathelicidins as Anticancer Therapy

Besides their role in inflammation, both cathelicidins and TLRs play an important role in the development and progression of cancer. TLR2 activation for instance, has been suggested as a possible therapeutic target, with local administration of a TLR2/6 agonist resulting in reduced tumor growth and prolonged survival in a pancreatic carcinoma mouse model (178). Alternatively, TLR9 activation by CpG-ODN has been shown to reduce metastasis and improve survival in pancreatic cancer (179) and neuroblastoma mouse models (180). As LL-37 has been shown to improve DNA-induced TLR9 activation, it might not be surprising that co-administration of CpG-ODN and LL-37 enhanced survival in a mouse ovarian-tumor model compared to CpG-ODN alone and that the LL-37-CpG-ODN combination enhanced the activation and proliferation of NK-cells, but not of T cells or macrophages, in the peritoneal cavity (181). On the other hand, inhibition of LPS-induced TLR4 activation reduces the migration and invasion capacity of the SW480 cancer cell line (182) and reduces pancreatic tumorigenesis in mice (183). While not specifically tested with cathelicidins, peptides such as LL-37 could also provide efficient inhibition of TLR4 and could thereby be of therapeutic value in tumors which have strongly enhanced TLR4 expression, for example numerous ovarian epithelial cancers (184). Furthermore, cathelicidins can also have beneficial anti-cancer effects in the absence of specific TLR ligands. In gastric or colon tumors, where the expression of LL-37 is reduced (185), treatment with LL-37 activates caspase-independent apoptosis and reduces tumor progression (186). Additionally, the application of CRAMP as adjuvant for HPV vaccination reduces tumor growth, albeit no direct anti-tumor effects of CRAMP were observed when used to treat HPV-induced tumors (175).

Nevertheless, overexpression of LL-37 has also been linked to increased tumor growth, enhanced invasiveness and bad prognosis in malignant melanomas and ovarian, lung, prostate and breast cancers, by stimulating the growth receptors of the EGFR and ERB-family (187). In addition, increased TLR expression in ovarian and pancreatic cancers is associated with poor clinical outcome (188, 189). Together, this shows that cathelicidins, either directly or through modulation of TLR activation, can be useful in the development of novel anti-cancer therapies, but that the potential negative effects of these peptides should not be overlooked. In this respect, modification of synthetic cathelicidin-like peptides could provide a solution to these issues, by structurally improving the peptide to increase desirable effects while at the same time limiting unwanted side effects.

## Conclusion and Outlook

The fact that cathelicidins are a highly conserved part of the innate immune system in vertebrates, together with their apparent multifunctional nature, has led to a large interest in these peptides across various disciplines, including e.g., microbiology, immunology, oncology and dermatology. This multidisciplinary approach has uncovered a wide variety of effects that these peptides can exert. However, the potency of these different effects can vary strongly. Among their immunoregulatory functions, the regulation of TLR activation can be counted among their more potent functions, with cathelicidins being able to strongly reduce LPS- or LTA- induced TLR activation and almost completely inhibiting TLR-mediated inflammatory responses when they kill bacteria. On the other hand, DNA can be transformed from a quickly degradable extracellular factor into a very potent inflammatory signal in the context of various auto-immune diseases. While direct interaction between cathelicidins and TLR ligands in most of these cases plays an important role in regulating TLR activation, more research is required to uncover the more complex direct effects of cathelicidins on signaling pathways such as the induction of autophagy. Autophagy can play a role in an enormous set of different intracellular pathways. Activation of autophagy by cathelicidins could also have a major impact on TLR signaling besides those effects mentioned in this review. Importantly, while cathelicidins are conserved among vertebrates, the amino acid composition and 3D structure of the mature peptides vary a lot. This means that one should be careful to extrapolate findings between different cathelicidins without supporting laboratory evidence.

Finally, the literature discussed in this review shows that cathelicidins could have strong prophylactic and therapeutic value aside from their antimicrobial activities. This includes dampening inflammation when treating infections to prevent sepsis as well as improving vaccine responses through an improved immune response against target antigens. In addition, it has been shown using structurally modified cathelicidins that certain functions can be enhanced or, alternatively, be limited. Thus, cathelicidin derivatives can be designed with specific therapeutic properties while limiting any unwanted side effects. Overall, it may be concluded that, despite the fact that cathelicidins have been discovered nearly 30 years ago, the elucidation of new properties and functions in recent years continues to provide more insight in the physiological roles and potential applications of cathelicidins.

## Author Contributions

MS and MC have written the review. RH designed the graphics. HH, EV, and RH, carefully read and corrected the text and wrote paragraphs of their expertise.

## Conflict of Interest

The authors declare that the research was conducted in the absence of any commercial or financial relationships that could be construed as a potential conflict of interest.
